# Bis[2-(3,4-disulfanylphen­yl)acetato]bis­(2-methyl-1*H*-imidazole-κ*N* ^3^)zinc(II)

**DOI:** 10.1107/S1600536809033893

**Published:** 2009-08-29

**Authors:** Qiang Wang, Li-Jun Wang, Jian Hou

**Affiliations:** a Engineering Research Center for Clean Production of Textile Dyeing and Printing, Ministry of Education, Wuhan 430073, People’s Republic of China

## Abstract

In the title mononuclear zinc(II) complex, [Zn(C_8_H_7_O_2_S_2_)_2_(C_4_H_6_N_2_)_2_], the Zn^II^ atom, lying on a twofold axis, is coordinated by two O atoms from two 2-(3,4-disulfanylphen­yl)acetate anions and by two N atoms from 2-methyl­imidazole ligands in a distorted tetra­hdral coordination. The crystal structure is stabilized by inter­molecular C—H⋯O and N—H⋯O hydrogen bonds and π–π inter­actions with a centroid–centroid distance of 3.6136 (16) Å.

## Related literature

For general background to organometallic complexes and their applications, see: Sommerfeldt *et al.* (2008 ▶); Huang *et al.* (2007 ▶); Neville *et al.* (2008 ▶). Zinc derivatives are of particular inter­est owing to their unique photosensitizing properties for photodynamic therapy, see: You *et al.* (2006 ▶); Shi *et al.* (2008 ▶); Xiao *et al.* (2008 ▶, 2009 ▶). For related structures, see: Yang *et al.* (2004 ▶); You *et al.* (2003 ▶, 2004 ▶); Qiu *et al.* (2004 ▶, 2007 ▶); Halcrow *et al.* (2000 ▶).
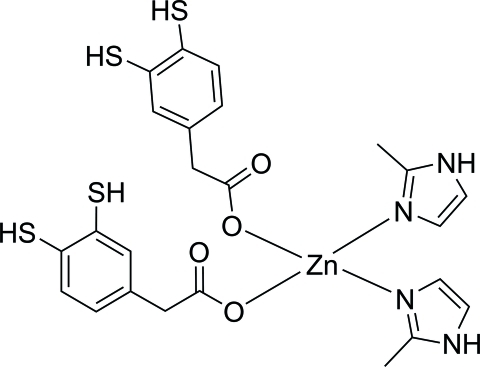


## Experimental

### 

#### Crystal data

[Zn(C_8_H_7_O_2_S_2_)_2_(C_4_H_6_N_2_)_2_]
*M* *_r_* = 628.16
Monoclinic, 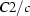

*a* = 12.9599 (9) Å
*b* = 9.3909 (6) Å
*c* = 21.5549 (12) Å
β = 91.579 (2)°
*V* = 2622.4 (3) Å^3^
*Z* = 4
Mo *K*α radiation
μ = 1.30 mm^−1^
*T* = 298 K
0.30 × 0.20 × 0.20 mm

#### Data collection

Bruker APEXII area-detector diffractometer
Absorption correction: multi-scan (*SADABS*; Sheldrick, 1996 ▶) *T*_min_ = 0.697, *T*_max_ = 0.782
15712 measured reflections
3260 independent reflections
3102 reflections with *I* > 2σ(*I*)
*R*_int_ = 0.020

#### Refinement

*R*[*F* ^2^ > 2σ(*F* ^2^)] = 0.041
*wR*(*F* ^2^) = 0.125
*S* = 1.12
3260 reflections
175 parameters
H atoms treated by a mixture of independent and constrained refinement
Δρ_max_ = 0.70 e Å^−3^
Δρ_min_ = −0.60 e Å^−3^

### 

Data collection: *SMART* (Bruker, 2000 ▶); cell refinement: *SAINT* (Bruker, 2000 ▶); data reduction: *SAINT*; program(s) used to solve structure: *SHELXS97* (Sheldrick, 2008 ▶); program(s) used to refine structure: *SHELXL97* (Sheldrick, 2008 ▶); molecular graphics: *SHELXTL* (Sheldrick, 2008 ▶); software used to prepare material for publication: *SHELXTL*.

## Supplementary Material

Crystal structure: contains datablocks global, I. DOI: 10.1107/S1600536809033893/rk2159sup1.cif

Structure factors: contains datablocks I. DOI: 10.1107/S1600536809033893/rk2159Isup2.hkl

Additional supplementary materials:  crystallographic information; 3D view; checkCIF report

## Figures and Tables

**Table 1:** Hydrogen-bond geometry (Å, °)

*D*—H⋯*A*	*D*—H	H⋯*A*	*D*⋯*A*	*D*—H⋯*A*
C12—H12*B*⋯O1^i^	0.96	2.46	3.381 (4)	161
N2—H2⋯O2^ii^	0.85 (4)	1.94 (4)	2.785 (3)	176 (4)

Symmetry codes: (i) 
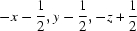
; (ii) 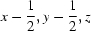.

**Fig. 1.**
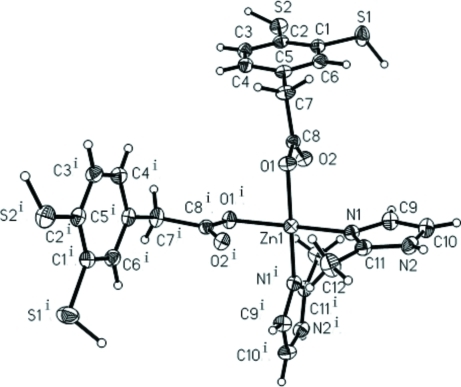
The asymmetric unit of I with the atom numbering scheme. Displacement ellipsoids are drawn at the 30% probability level. H atoms are presented as a small spheres of arbitrary radius.

**Fig. 2.**
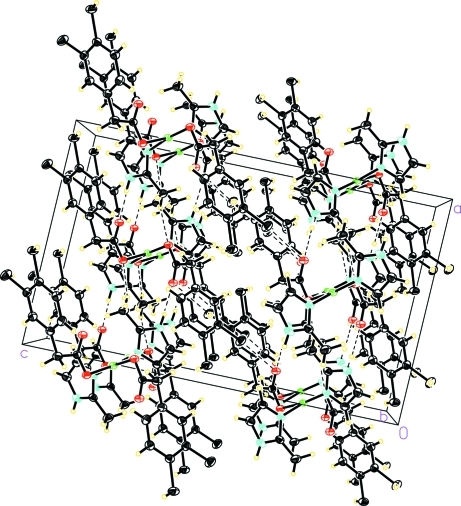
A network is formed through C–H···O and N–H···O intermolecular hydrogen bonds. Dashed lines indicate hydrogen bonds.

## supplementary crystallographic information

### Comment

Numerous organometallic complexes have been designed for a number of potential applications, such as in synthetic chemistry (Sommerfeldt *et al.*, 2008), as luminescence materials (Huang *et al.*, 2007) and as magnetic materials (Neville *et al.*, 2008). Zinc derivatives are particularly interesting owing to their unique photosensitizing properties for photodynamic therapy (You *et al.*, 2006; Shi *et al.*, 2008; Xiao *et al.*, 2008; Xiao *et al.*, 2009), magnetic circularly polarized luminescence and magnetic circular dichroism spectra. We have reported the structures of a few zinc(II) complexes (Yang *et al.*, 2004; You *et al.*, 2003, 2004; Qiu *et al.*, 2007). As an extension of our work on the structural characterization of zinc compounds, we report the crystal structure of the title compound, (I), which has been determined in an attempt to understand the structural behaviour of sulfur containing ligands when coordinating to zinc carboxylates.

The present X-ray single-crystal diffraction study reveals that I, bis(3,4-dimercaptophenylaceto)-bis(2-methylimidazole-kappa*N*^3^)-zinc, consists of a Zn atom, two 3,4-dimercaptophenylaceto ligands and two 2-methylimidazole ligands. As shown on Fig. 1, the central Zn atom exhibits 4-coordination by two N atoms of position 3 from two imidazole molecules respectively and two O atoms from two 2-(3,4-disulfanylphenyl)acetate anions, forming a slightly distorted square plane. The distortion arises from the N1–Zn1–O1^i^ axis, which is not perfectly standing in a line. The Zn–O distances is 1.9858 (18)Å is in accord with similar distance reported previously (You *et al.*, 2004; Qiu *et al.*, 2004). The Zn–N distance of 1.972 (2)Å is slightly shorter than other reported distances (Halcrow *et al.*, 2000).

The H-bonding interactions occur between the N–H in imidazole as well as the methyl group (C12–H12B) as donors and O2 together with O1 atom of the carboxyl group as acceptors (Fig. 2, Table 1). These intermolecular H-bonds construct an infinite network. Concurrently, the network are solidated by weak intermolecular π-π interactions between C1-C6 ring and N1/C9/C10/N2/C11 rings with centre to centre distance of 3.6136 (16)Å.

### Experimental

The 0.5 mmol of zinc oxide, 1 mmol of 3,4-dimercaptophenylacetic acid and 1 mmol of 2-methylimidazole were dissolved in 10 ml methanol. The result solution was heated to 423 K for 12 h. The reactor was cooled to room temperature at a rate of 10 K h^-1^. The mixture was filtered and held at room temperature for 8 d. Colourless block crystals were isolated in 32% yield.

### Refinement

The H atom bonded to N1 was located in a difference Fourier map and refined freely. All other H atoms were placed in geometrically idealized positions and constrained to ride on their parent atoms with C–H of 0.93Å for the aromatic H atoms, 0.96Å for the CH_3_ groups and S–H of 1.20Å. *U*_iso_(H) values were set at 1.2 times *U*_eq_(C) for aromatic H, 1.5 times *U*_eq_(C) for CH_3_ and 1.5 times *U*_eq_(S) for S–H groups.

### Crystal data

**Table:**

[Zn(C_8_H_7_O_2_S_2_)_2_(C_4_H_6_N_2_)_2_]	*F*(000) = 1296
*M**_r_* = 628.16	*D*_x_ = 1.591 Mg m^−^^3^
Monoclinic, *C*2/*c*	Mo *K*α radiation, λ = 0.71073 Å
Hall symbol: -C 2yc	Cell parameters from 3063 reflections
*a* = 12.9599 (9) Å	θ = 2.3–26.7°
*b* = 9.3909 (6) Å	µ = 1.30 mm^−^^1^
*c* = 21.5549 (12) Å	*T* = 298 K
β = 91.579 (2)°	Block, colourless
*V* = 2622.4 (3) Å^3^	0.30 × 0.20 × 0.20 mm
*Z* = 4	

### Data collection

**Table:**

Bruker APEXII area-detector diffractometer	3260 independent reflections
Radiation source: fine-focus sealed tube	3102 reflections with *I* > 2σ(*I*)
graphite	*R*_int_ = 0.020
φ and ω scans	θ_max_ = 28.3°, θ_min_ = 1.9°
Absorption correction: multi-scan (*SADABS*; Sheldrick, 1996)	*h* = −17→17
*T*_min_ = 0.697, *T*_max_ = 0.782	*k* = −12→12
15712 measured reflections	*l* = −28→28

### Refinement

**Table:**

Refinement on *F*^2^	Primary atom site location: structure-invariant direct methods
Least-squares matrix: full	Secondary atom site location: difference Fourier map
*R*[*F*^2^ > 2σ(*F*^2^)] = 0.041	Hydrogen site location: inferred from neighbouring sites
*wR*(*F*^2^) = 0.125	H atoms treated by a mixture of independent and constrained refinement
*S* = 1.12	*w* = 1/[σ^2^(*F*_o_^2^) + (0.0644*P*)^2^ + 4.9284*P*] where *P* = (*F*_o_^2^ + 2*F*_c_^2^)/3
3260 reflections	(Δ/σ)_max_ = 0.001
175 parameters	Δρ_max_ = 0.70 e Å^−^^3^
0 restraints	Δρ_min_ = −0.60 e Å^−^^3^

### Special details

**Table:**

Geometry. All s.u.'s (except the s.u. in the dihedral angle between two l.s. planes) are estimated using the full covariance matrix. The cell s.u.'s are taken into account individually in the estimation of s.u.'s in distances, angles and torsion angles; correlations between s.u.'s in cell parameters are only used when they are defined by crystal symmetry. An approximate (isotropic) treatment of cell s.u.'s is used for estimating s.u.'s involving l.s. planes.
Refinement. Refinement of *F*^2^ against ALL reflections. The weighted *R*-factor *wR* and goodness of fit *S* are based on *F*^2^, conventional *R*-factors *R* are based on *F*, with *F* set to zero for negative *F*^2^. The threshold expression of *F*^2^ > σ(*F*^2^) is used only for calculating *R*-factors(gt) *etc*. and is not relevant to the choice of reflections for refinement. *R*-factors based on *F*^2^ are statistically about twice as large as those based on *F*, and *R*-factors based on ALL data will be even larger.

### Fractional atomic coordinates and isotropic or equivalent isotropic displacement parameters (Å^2^)

**Table:**

	*x*	*y*	*z*	*U*_iso_*/*U*_eq_	
C1	0.2396 (2)	0.5043 (3)	0.48533 (12)	0.0379 (5)	
N1	−0.07005 (15)	−0.0094 (2)	0.29964 (10)	0.0336 (4)	
O1	−0.05381 (13)	0.28914 (19)	0.30382 (8)	0.0365 (4)	
S1	0.28934 (7)	0.42402 (10)	0.55206 (4)	0.0571 (2)	
H3A	0.2872	0.2970	0.5459	0.086*	
Zn1	0.0000	0.13605 (4)	0.2500	0.03067 (13)	
C2	0.2944 (2)	0.6098 (3)	0.45584 (13)	0.0364 (5)	
H2	−0.251 (3)	−0.178 (4)	0.3375 (18)	0.058 (10)*	
N2	−0.18947 (19)	−0.1506 (2)	0.33454 (12)	0.0412 (5)	
O2	0.10600 (13)	0.25971 (19)	0.33887 (9)	0.0366 (4)	
S2	0.41436 (6)	0.66341 (9)	0.48428 (4)	0.0489 (2)	
H3B	0.4310	0.7829	0.4676	0.073*	
C3	0.2536 (2)	0.6715 (3)	0.40205 (13)	0.0403 (6)	
H3	0.2896	0.7431	0.3821	0.048*	
C4	0.1594 (2)	0.6260 (3)	0.37831 (13)	0.0415 (6)	
H4	0.1317	0.6689	0.3427	0.050*	
C5	0.10476 (19)	0.5171 (3)	0.40649 (12)	0.0361 (5)	
C6	0.1456 (2)	0.4587 (3)	0.46066 (12)	0.0393 (5)	
H6	0.1093	0.3877	0.4808	0.047*	
C7	0.0069 (2)	0.4586 (3)	0.37719 (14)	0.0422 (6)	
H7A	−0.0251	0.5320	0.3515	0.051*	
H7B	−0.0406	0.4356	0.4097	0.051*	
C8	0.02273 (18)	0.3261 (3)	0.33751 (11)	0.0312 (4)	
C9	−0.0334 (2)	−0.0732 (3)	0.35314 (13)	0.0417 (6)	
H9	0.0316	−0.0587	0.3714	0.050*	
C10	−0.1071 (2)	−0.1607 (3)	0.37499 (15)	0.0449 (6)	
H10	−0.1024	−0.2166	0.4106	0.054*	
C11	−0.16510 (19)	−0.0582 (3)	0.28945 (12)	0.0370 (5)	
C12	−0.2349 (2)	−0.0175 (4)	0.23679 (16)	0.0590 (9)	
H12A	−0.2573	0.0791	0.2420	0.088*	
H12B	−0.2938	−0.0795	0.2355	0.088*	
H12C	−0.1987	−0.0257	0.1987	0.088*	

### Atomic displacement parameters (Å^2^)

**Table:**

	*U*^11^	*U*^22^	*U*^33^	*U*^12^	*U*^13^	*U*^23^
C1	0.0419 (13)	0.0362 (12)	0.0355 (12)	−0.0028 (10)	−0.0015 (10)	−0.0026 (10)
N1	0.0279 (9)	0.0342 (10)	0.0390 (10)	−0.0042 (8)	0.0047 (8)	−0.0016 (8)
O1	0.0281 (8)	0.0389 (9)	0.0421 (9)	0.0006 (7)	−0.0039 (7)	−0.0082 (7)
S1	0.0623 (5)	0.0593 (5)	0.0487 (4)	−0.0145 (4)	−0.0168 (4)	0.0159 (4)
Zn1	0.0238 (2)	0.0328 (2)	0.0354 (2)	0.000	0.00027 (14)	0.000
C2	0.0337 (12)	0.0335 (11)	0.0419 (13)	−0.0027 (9)	0.0013 (10)	−0.0070 (10)
N2	0.0345 (11)	0.0364 (11)	0.0533 (13)	−0.0079 (9)	0.0095 (10)	0.0012 (9)
O2	0.0286 (8)	0.0360 (9)	0.0449 (9)	0.0012 (7)	−0.0033 (7)	−0.0036 (7)
S2	0.0354 (4)	0.0513 (4)	0.0595 (4)	−0.0100 (3)	−0.0046 (3)	−0.0066 (3)
C3	0.0433 (14)	0.0345 (12)	0.0433 (14)	−0.0051 (10)	0.0040 (11)	0.0005 (10)
C4	0.0476 (15)	0.0379 (13)	0.0387 (13)	0.0003 (11)	−0.0022 (11)	−0.0006 (10)
C5	0.0334 (11)	0.0335 (12)	0.0413 (12)	−0.0001 (9)	0.0012 (10)	−0.0105 (10)
C6	0.0409 (13)	0.0356 (12)	0.0416 (13)	−0.0060 (10)	0.0032 (10)	−0.0028 (10)
C7	0.0337 (12)	0.0407 (13)	0.0519 (15)	0.0020 (10)	−0.0028 (11)	−0.0135 (12)
C8	0.0306 (11)	0.0303 (10)	0.0327 (11)	−0.0026 (9)	0.0011 (9)	−0.0005 (9)
C9	0.0318 (12)	0.0472 (15)	0.0461 (14)	0.0008 (10)	0.0006 (10)	0.0051 (12)
C10	0.0420 (14)	0.0423 (14)	0.0507 (15)	0.0018 (11)	0.0056 (12)	0.0103 (12)
C11	0.0317 (11)	0.0370 (12)	0.0424 (13)	−0.0046 (9)	0.0055 (10)	−0.0045 (10)
C12	0.0418 (16)	0.081 (2)	0.0540 (18)	−0.0166 (16)	−0.0088 (13)	0.0081 (17)

### Geometric parameters (Å, °)

**Table:**

C1—C6	1.384 (4)	C3—C4	1.379 (4)
C1—C2	1.384 (4)	C3—H3	0.9300
C1—S1	1.732 (3)	C4—C5	1.392 (4)
N1—C11	1.327 (3)	C4—H4	0.9300
N1—C9	1.373 (3)	C5—C6	1.382 (4)
N1—Zn1	1.972 (2)	C5—C7	1.506 (3)
O1—C8	1.262 (3)	C6—H6	0.9300
O1—Zn1	1.9858 (18)	C7—C8	1.527 (3)
S1—H3A	1.2000	C7—H7A	0.9700
Zn1—N1^i^	1.972 (2)	C7—H7B	0.9700
Zn1—O1^i^	1.9858 (18)	C9—C10	1.354 (4)
C2—C3	1.388 (4)	C9—H9	0.9300
C2—S2	1.730 (3)	C10—H10	0.9300
N2—C11	1.347 (4)	C11—C12	1.482 (4)
N2—C10	1.363 (4)	C12—H12A	0.9600
N2—H2	0.85 (4)	C12—H12B	0.9600
O2—C8	1.246 (3)	C12—H12C	0.9600
S2—H3B	1.2000		
			
C6—C1—C2	120.2 (2)	C4—C5—C7	121.2 (3)
C6—C1—S1	119.2 (2)	C5—C6—C1	121.0 (2)
C2—C1—S1	120.6 (2)	C5—C6—H6	119.5
C11—N1—C9	106.7 (2)	C1—C6—H6	119.5
C11—N1—Zn1	126.04 (18)	C5—C7—C8	114.0 (2)
C9—N1—Zn1	127.18 (17)	C5—C7—H7A	108.7
C8—O1—Zn1	104.61 (15)	C8—C7—H7A	108.7
C1—S1—H3A	109.5	C5—C7—H7B	108.7
N1—Zn1—N1^i^	92.29 (12)	C8—C7—H7B	108.7
N1—Zn1—O1	90.59 (8)	H7A—C7—H7B	107.6
N1^i^—Zn1—O1	173.13 (8)	O2—C8—O1	122.9 (2)
N1—Zn1—O1^i^	173.13 (8)	O2—C8—C7	121.6 (2)
N1^i^—Zn1—O1^i^	90.59 (8)	O1—C8—C7	115.5 (2)
O1—Zn1—O1^i^	87.23 (11)	C10—C9—N1	109.0 (2)
C1—C2—C3	119.6 (2)	C10—C9—H9	125.5
C1—C2—S2	120.9 (2)	N1—C9—H9	125.5
C3—C2—S2	119.5 (2)	C9—C10—N2	106.4 (3)
C11—N2—C10	108.1 (2)	C9—C10—H10	126.8
C11—N2—H2	120 (3)	N2—C10—H10	126.8
C10—N2—H2	131 (3)	N1—C11—N2	109.8 (2)
C2—S2—H3B	109.5	N1—C11—C12	125.6 (3)
C4—C3—C2	119.6 (2)	N2—C11—C12	124.6 (2)
C4—C3—H3	120.2	C11—C12—H12A	109.5
C2—C3—H3	120.2	C11—C12—H12B	109.5
C3—C4—C5	121.4 (3)	H12A—C12—H12B	109.5
C3—C4—H4	119.3	C11—C12—H12C	109.5
C5—C4—H4	119.3	H12A—C12—H12C	109.5
C6—C5—C4	118.2 (2)	H12B—C12—H12C	109.5
C6—C5—C7	120.5 (2)		
			
C11—N1—Zn1—N1^i^	−96.2 (2)	C6—C5—C7—C8	−81.8 (3)
C9—N1—Zn1—N1^i^	88.2 (2)	C4—C5—C7—C8	94.8 (3)
C8—O1—Zn1—O1^i^	−82.04 (15)	Zn1—O1—C8—O2	−8.8 (3)
C6—C1—C2—C3	1.2 (4)	Zn1—O1—C8—C7	171.33 (18)
S1—C1—C2—C3	179.3 (2)	C5—C7—C8—O2	12.1 (4)
C6—C1—C2—S2	−177.5 (2)	C5—C7—C8—O1	−168.1 (2)
S1—C1—C2—S2	0.6 (3)	C11—N1—C9—C10	0.1 (3)
C1—C2—C3—C4	−0.6 (4)	Zn1—N1—C9—C10	176.3 (2)
S2—C2—C3—C4	178.1 (2)	N1—C9—C10—N2	0.1 (3)
C2—C3—C4—C5	−1.2 (4)	C11—N2—C10—C9	−0.2 (3)
C3—C4—C5—C6	2.4 (4)	C9—N1—C11—N2	−0.2 (3)
C3—C4—C5—C7	−174.3 (2)	Zn1—N1—C11—N2	−176.49 (17)
C4—C5—C6—C1	−1.8 (4)	C9—N1—C11—C12	179.6 (3)
C7—C5—C6—C1	174.9 (2)	Zn1—N1—C11—C12	3.3 (4)
C2—C1—C6—C5	0.0 (4)	C10—N2—C11—N1	0.2 (3)
S1—C1—C6—C5	−178.1 (2)	C10—N2—C11—C12	−179.6 (3)

Symmetry codes: (i) −*x*, *y*, −*z*+1/2.

### Hydrogen-bond geometry (Å, °)

**Table:**

*D*—H···*A*	*D*—H	H···*A*	*D*···*A*	*D*—H···*A*
C12—H12B···O1^ii^	0.96	2.46	3.381 (4)	161
N2—H2···O2^iii^	0.85 (4)	1.94 (4)	2.785 (3)	176 (4)

Symmetry codes: (ii) −*x*−1/2, *y*−1/2, −*z*+1/2; (iii) *x*−1/2, *y*−1/2, *z*.
